# A novel hybrid model to predict concomitant diseases for Hashimoto’s thyroiditis

**DOI:** 10.1186/s12859-023-05443-5

**Published:** 2023-08-24

**Authors:** Pınar Karadayı Ataş

**Affiliations:** Department of Computer Engineering, Istanbul Arel University, 34537 Buyukcekmece, Istanbul Turkey

**Keywords:** Hasimoto’s thyroid, Autoimmun disease, Concominant disease, Machine learning, Hybrid classification

## Abstract

Hashimoto’s thyroiditis is an autoimmune disorder characterized by the destruction of thyroid cells through immune-mediated mechanisms involving cells and antibodies. The condition can trigger disturbances in metabolism, leading to the development of other autoimmune diseases, known as concomitant diseases. Multiple concomitant diseases may coexist in a single individual, making it challenging to diagnose and manage them effectively. This study aims to propose a novel hybrid algorithm that classifies concomitant diseases associated with Hashimoto’s thyroiditis based on sequences. The approach involves building distinct prediction models for each class and using the output of one model as input for the subsequent one, resulting in a dynamic decision-making process. Genes associated with concomitant diseases were collected alongside those related to Hashimoto’s thyroiditis, and their sequences were obtained from the NCBI site in fasta format. The hybrid algorithm was evaluated against common machine learning algorithms and their various combinations. The experimental results demonstrate that the proposed hybrid model outperforms existing classification methods in terms of performance metrics. The significance of this study lies in its two distinctive aspects. Firstly, it presents a new benchmarking dataset that has not been previously developed in this field, using diverse methods. Secondly, it proposes a more effective and efficient solution that accounts for the dynamic nature of the dataset. The hybrid approach holds promise in investigating the genetic heterogeneity of complex diseases such as Hashimoto’s thyroiditis and identifying new autoimmune disease genes. Additionally, the results of this study may aid in the development of genetic screening tools and laboratory experiments targeting Hashimoto’s thyroiditis genetic risk factors. New software, models, and techniques for computing, including systems biology, machine learning, and artificial intelligence, are used in our study.

## Introduction

Autoimmune thyroid diseases (AITD) are one of the most common health problems in the world. Autoimmune thyroid diseases include two diseases, Graves’ disease (GD) and Hashimoto’s thyroiditis (HT). They affect 2–5$\%$ of the Western population [1, 2]. Thyroid autoimmunity is a combination of environmental, genetic, and endogenous factors. The various clinical symptoms include infiltration of T and B lymphocytes in the thyroid gland and production of thyroid autoantibodies [3]. Autoimmune thyroid diseases may be associated with other autoimmune thyroid diseases. Other autoimmune diseases that may be concomitant include diseases such as rheumatoid arthritis, vitiligo, myasthenia gravis, celiac disease, and primary adrenal insufficiency [4]. The prevalence of autoimmune thyroid disease (ATD) disorders is increasing day by day, and ATD disorders are more common in women. Aging contributes to the development of hypothyroidism. Prevalence and incidence may vary geographically, and the frequency of antibodies may differ among populations. Many environmental factors such as iodine, radiation, smoking, infections, stress, drugs, and medications have been implicated in ATD [5, 6].

Autoimmune disease is a complex disease involving numerous biological interactions at various physical and biological levels [7]. Therefore, the complexity can lead to cellular and physiological systematic problems. Computational and bioinformatics models contribute to the development of new biological discoveries and clinical medicine. The development of computational data enables rapid experimental and analytical modeling, resulting in information-rich biological data with high throughput [8, 9].

The use of a hybrid model for classification, which combines multiple methods, can indeed have many potential applications in computational tools for strategic decision support systems. By combining the strengths of different methods, the hybrid model can improve the accuracy and reliability of the classification results. For example, in the field of healthcare, a hybrid model can be used to classify medical data and assist with diagnosis and treatment planning. Overall, the implementation of a hybrid model in computational tools can provide decision-makers with more accurate and comprehensive information, which can help them make better-informed decisions in a variety of industries and contexts. In this study, The novel hybrid model approach for classification, involving the combination of multiple methods, exhibits promising potential for application in computational tools designed to support strategic decision-making systems.

The development of biotechnology and data analysis methods in biology has led to a new field called bioinformatics, while the progress in machine learning technology in biology has produced many useful algorithms that are scalable and effective. The problem has shifted from gathering data to gaining knowledge from data, and both sides of this coin have had significant developments in recent years. The connection between bioinformatics and machine learning (ML) can be a bridge to help analyze biomedical data. There are some questions that need to be addressed, which could help spur the development of strong machine learning algorithms in bioinformatics.ML models have advantages over conventional statistical approaches, such as nonlinear pattern identification, less biased auto-learning, and greater flexibility to prevent overfitting [10]. Data-driven ML models can forecast Medication Adherence (MA) by using specific features from patients past medical information. These models can help with treatment planning, predict negative outcomes, and speed up the management of perioperative healthcare. ML approaches are suitable for finding complex connections between patient characteristics and MA, according to Ref. [11]. Saving time on time-consuming clinical tasks can be achieved by employing ML algorithms to find elevant features for predictive analytics.

In the study by Zhongzhi et al. [12], 488 patients who had been given the diagnosis of papillary thyroid cancer (PTC) by ultrasound-guided fine-needle aspiration biopsy were included. Clinicopathological information was gathered, and using univariate analysis and binary logistic regression, the correlation between central lymph node metastasis (CLNM) and clinicopathological features was examined. Prediction models were then created. Circular graphs (DAWGs) were suggested by Levy and Stormo [13] as a method of classifying DNA sequences. To categorize DNA sequences, Muller and Koonin [14] proposed using vector space. A multi-classifier method was put up by Ranawana and Palade (2005) to recognize DNA sequences that contain E. coli promoter sequences. He uses the sequence to train four separate neural networks after encoding it using four different coding techniques. A variation of the logarithmic opinion pool method was utilized in the aggregation function to integrate the classification outcomes of the four distinct neural networks. According to experiments, the neural network can produce somewhat varied outputs when the same input is fed to it using various encoding techniques. In addition, we can get results that are superior to the neural network’s single performance when the output of multiple classifiers trained on the same input data are combined into a multi-classifier. The difficulty in obtaining the appropriate neural network parameters is the fundamental drawback of the neural network design. The neural network will be put into use, and the encoding technique will be optimized [15]. In order to determine the DNA sequence classification of E. coli promoters, Ma et al. [16] presented a DNA sequence classification based on the union of the expectation-maximization method with a neural network. The 35 and 10 binding sites in the E. coli promoter sequence are located by Ma Q using an enhanced expectation-maximization technique. The distribution of the lengths of the spacers between binding sites and between binding sites and transcription start sites is no longer taken for granted. The probability distribution of these lengths is instead. A variable-order hidden Markov model with the continuous state: VOGUE was proposed by Zaki et al. in 2010. VOGUE builds a variable sequence hidden Markov model after using a variable sequence mining technique to identify frequent patterns with varying lengths and spacings between elements [17]. The classification accuracy of VOGUE is higher than that of conventional HMM. However, the frequency statistical properties of the sub-sequences in the sequence are not taken into account, which has an impact on the model’s capacity for generalization.

Feature extraction is a prerequisite for the machine learning technique used for supervised learning classification tasks. Two distinct deep learning models were suggested by Bosco and Di Gangi [18]. On five datasets, they performed classification tasks using the model. It turns out that deep learning models or neural networks are capable of automatically extracting useful characteristics from input patterns.

The characterization and annotation of sequences is a significant challenge in genomics. Many machine learning methods have been used to this issue in recent years. In any event, the feature selection process continues to be the main challenge underlying the issue. There are no distinguishing characteristics in the series. Additionally, high-dimensional issues are easily introduced by the general representation method. The challenge of study is how to efficiently represent sequence attributes and analyze high dimensional data [19].

To identify and categorize the cases affected by cancer disease, [20] used a hybrid algorithm in the research that combined the particle swarm optimization (PSO) algorithm and a machine learning method. Motieghader et al. [21] suggested the GALA mixed hybrid algorithm, which combined a genetic algorithm (GA) and a machine learning strategy.

It should be noted that when applied to complex and high-dimensional data, such as biomedical datasets, standard algorithms and even ensemble learning methods for classification problems face significant challenges, particularly in terms of computational efficiency and effectiveness. Therefore, we propose a novel hybrid method to address these issues and enhance the performance of the classification algorithm. The hybrid algorithm combines machine learning techniques, such as clustering and classification, to improve accuracy and efficiency. Our approach is distinct from ensemble methods [22–24], which integrate decisions, as we use the results of one algorithm as the input for another throughout.

In this study, A new dataset of autoimmune diseases associated with Hashimoto’s thyroid and related genes by utilizing various database sites were generated, such as PUBMED and OMIM. The experimental results demonstrate that our proposed approach performs favourably. By analyzing structural and functional data using machine learning and bioinformatics tools, we seek to understand the relationship between thyroid autoimmune diseases and other concurrent diseases using our novel hybrid classification model.

## Materials and method

### Dataset preperation procedure

DNA is an organic macromolecule that is the basic storage unit of genetic information and was significantly progressed in research in the 1980s. Since the development of genome sequencing systems, there has been a shift in focus from collecting original data, to interpreting data. Databases of Online Mendelian Inheritance in Man (OMIM) [25], National Library of Medicine (PUBMED) [26], Entrez Gene on National Center for Biological Information (NCBI) [27], NCBI dbSNP [28], and SWISS Prot databases [29] were used to obtained information for autoimmune thyroid diseases and concomitant diseases. While doing our research, we searched articles from PUBMED and related genes were screened. These genes were then analyzed for accuracy in the OMIM database. The sequences of the genes were taken from the NCBI site in Fasta format and processed in machine learning. We used these databases while conducting our research because they are the most used, reliable and free databases.

Autoimmune codominant diseases in Hashimoto thyroid patients are Myasthenia gravis, Vitiligo, Autoimmune hepatitis, Pemphigus, Rheumatoid arthritis, Type 1 diabetes, Lupus, Addisons disease, Graves disease, Premature ovarian failure, Pernicious anemia, Thrombocytopenic purpura, Ulcerative colitis, Autoimmune colitis, Autoimmune colitis Chronic autoimmune gastritis, SpondyloArthritis, Sjogren’s syndrome, Celiac disease, Alopecia areata. 62 genes associated with these diseases have been found. 21 of these 62 genes were found to be common with Hashimoto’s thyroid. This dataset has never been studied before and it took a long time to collect it. In addition to these genes, we have found one-hundred genes that are related to these 62 genes. The study comprehensively addresses the autoimmune codominant diseases under consideration, the relevant genes, and the detailed information regarding the sources from which the data was collected in Table 1.

**Table 1:** Diseases and genes associated with Hashimoto’s thyroiditis

Disease	Gene	Reference
Myasthenia gravis	PTPN22, CTLA4	Lin et al. [30], Lopomo and Berrih-Aknin [31]
Vitiligo	TYR, TG, TSHR, AIS1, forkhead transcription factor D3 (FOXD3), PTPN22 1858T (rs2476601), FOXP3 mutations TG/SLA	Cojocaru et al. [32], Said Fernandez et al. [33], Czajkowski [34]
Rheumatoid arthritis	PTPN22 (rs2476601), HLA-DR, B1-Arg74, CTLA4, PTPN22, FCRL3, IL2RA, BTG1, FCRL3	Yamamoto et al. [35], Lazurova et al. [36]
Type 1 diabetes	HLA-DRB1-03:01, HLA-DRB1-03:02, HLA-DRB1-04:01, HLA-DQA1-03:01, HLA-DQA1-05:01, HLA-DQB1-02:01, HLA-DQB1-03:01, HLA-DQB1-03:02. HLA-DRB1-03:01-DQA1-05:01-DQB1-02:01, HLA-DRB1-04:01-DQA1-03:01-DQB1-03:01, HLA-DRB1-04:01-DQA1-03:01-DQB1-03:02, CTLA4 (c.+6230GA, rs3087243), CTLA4 (c.49AG, rs231775), PTPN22 (c.+1858 CT, rs2476601), PTPN22 (rs2476601), IL2Ra (c.AG rs10795791),VDR, Bsm I rs1544410; Apa I rs7975232, Taq I rs731236, tumor necrosis factor (TNF, c.-863GA, rs1800630), C-type lectin domain containing 16 (CLEC16A) (rs12708716), erb-B2 receptor tyrosine kinase 3 (ERBB3) gene (rs2292399), the interferon induced with helicase C domain 1 (IFIH1) gene (rs1990760), CTLA4, PTPN22, IL2RA, CLEA16A, ERBB3, CCR5, CD247, VDR, NAA25, STAT4, INS, CAPSL, CD226 and IFIH1	Frommer and Kahaly [37], Baldini et al. [38]
Lupus	TPN22 (rs2476601)	Criswell et al. [39]
Graves’ disease	CTLA-4, PTPN22, HLA-DR3, TSHR, TG, HLA-DR$\beta$1, FOXP3, CD40, IL2RA	Tomer [40], Davies et al. [41]
Pernicious anemia	HLA-B8, DR3, DR5	Zulfiqar and Andres [42]
Sjogren’s syndrome	HLA-DR3 (DRB1:03:01)	Manuel et al. [43]
Celiac disease	HLA-DQ2 and HLA-DQ8 haplotypes, CTLA-4, CCR5	Mikosch et al. [44], de Carvalho and Fighera [45]
Hashimoto’s thyroids	CD25, CD40, FOXP3, CTLA4, PTPN22, thyroid stimulating hormone receptor, thyroglobulin, HLA-DR3, DRB1*04-DQB1*0301, HLA-DR B1-Arg74, CTLA4 gene +49A/G and CT 60, FoxP3, FoxP’s 2383CC polimorfizm, FOXE1, VAV3, CAPZB, PDE8B, TRIP2, LPP, FAM76B, RNASET2, CCR5, BACH2, ZFAT, SLC26A4, SESN3, DR5, DQ7 (HLA DQB1*0301, HLA DQB1*0304), DQw7, DRB1*04, DRB4*0101, HLA-A2, DRw53, VDR, TGF-beta, IFN-gamma, CYP27B1, IP6K3	Mikosch et al. [44], Frommer and Kahaly [37], Zaletel and Gaberscek [46], Kherrour et al. [47]

### Feature extraction

Nucleic acid composition, autocorrelation, and pseudo-nucleotide composition can be categorized as methods of extracting properties of DNA sequences. By counting the frequencies of occurrence of the nearest or non-contiguous residues along a DNA sequences, short-range or local sequence order information can be captured by methods in the nucleic acid composition category. The simplest method in this category is used as k-mer [48]. Here k represents the length of the substring in S. The frequencies of formation of k-mers are DNA sequences. The reverse complementary k-mer as a k-mer variant also references the principle of complementary base pairing. For instance;

1. Encoding DNA sequences using k-mers:
  Let’s consider a DNA sequence (ATCGATCGATCG); If we choose a k-mer size of 3 (k = 3), all possible 3-mers or trigrams can be extracted.
  Each of these 3-mers represents a small subsequence of the original DNA sequence.
2. Generating k-mer frequency vectors:
  The frequency vector we construct after extracting the k-mers will show where in the DNA sequence each k-mer appears. It would be “ATC”, “TCG”, “CGA”, “GAT”, “ATC”, “TCG”.
3. Extracting sequence properties using k-mers:
  K-mers can be used to extract various sequence properties or features from DNA sequences.
  One illustration is the k-mer composition, which shows how many k-mers are present in a sequence. Insights regarding the general complexity or patterns of the sequence can be gained from this.
4. Machine learning applications:
  In a variety of bioinformatics applications, K-mer representations can be employed as input features for machine learning methods.
  For instance, in the classification of DNA sequences, the sequences can be encoded using k-mers, and the feature vectors that arise can be fed into a machine learning model for classification or prediction tasks.

We tested the more popular 1-mer, 2-mer, 3-mer, 5-mer, and 6-mer. We ultimately decided that the 3-mer was the optimal parameter.

### Data preprocessing

#### Dimensionality reduction

Techniques for reducing the number of input variables in a dataset are referred to as dimensionality reduction. The curse of dimensionality, which is more commonly known, describes how adding more input features frequently makes it harder to model a predictive modeling problem. Data visualization frequently makes use of dimensionality reduction techniques and high-dimensional statistics.

A dimensionality reduction method that is frequently used for supervised classification issues is linear discriminant analysis(LDA), also known as normal discriminant analysis or discriminant function analysis. It is used to represent group differences by dividing groups into two or more classes. The features in a higher dimension space are projected into a lower dimension space using this technique [49]. LDA uses two criteria to develop a new axis. The first is to maximize the difference between the two classes’ means. second: Reduce variation within each class to a minimum.

#### Normalization

Feature scaling is data preprocessing to improve the performance of machine learning algorithms [50]. Normalization and standardization are the two most commonly used feature scaling techniques in machine learning. Normalization rescales the values to the range (0, 1), and standardization rescales the data so that the mean is 0 and the standard deviation is 1 [51]. In our study, min-max normalization was applied since normalization performed well compared to the comparison. The initial data are linearly processed before being used in the Min-max algorithm. The minimum and maximum values of a variable in the samples are denoted by the letters x*min* and x*max*. Since the distrubition of the attribute values in the dataset are equal to each other, min-max normalization algorithm is used rather than z-score.

Overall, min-max normalization was chosen over alternative approaches due to the following considerations: it properly normalized the data, preserved the integrity of the distribution, and matched the precise specifications [52–54]. Other benefits are Relative attribute distributions, the lack of outliers, and compatibility with pertinent analysis methodologies. Additionally, it produces desired results in relation to the benchmark dataset as we were able to maintain the dataset’s original distributional properties which makes it easier to conduct accurate analysis and reliable performance evaluation in the context of benchmarking.

Using linear mapping, the Min-max technique scales a variable in the training samples from [xmin, xmax] to [$-1$, 1] (or [0, 1]). However, the scaled values will be outside the bounds of the interval [$-1$, 1] (or [0, 1]) when the unseen testing samples fall outside the training data range of the variable, and that could cause issues in some applications. In addition, it is very sensitive to outliers, as shown in the following sections.

### Baseline classification algorithms

For the proposes of DNA classification issues, frequently used techniques in the litreture are Support Vector Machines (SVM) [55–57], Random Forest [58–60], Logistic Regression [61–63], and K-Nearest Neighbors (KNN) [53, 64]. The SVM algorithm creates an ideal hyperplane to divide various classes by maximizing the margin. On the other hand, Random Forest is an ensemble method that effectively manages high-dimensional data by combining many decision trees to produce predictions. A popular linear model is logistic regression which employs a logistic function to assess the probabilities of various classes. Lastly, the KNN algorithm is excellent for DNA classification jobs since it classifies data points based on their proximity to the k nearest neighbors. These algorithms were selected for the study because of their shown efficacy and adaptability in handling DNA sequences, which provides a strong platform for comparison and evaluation.

### The proposed hybrid classification method

To enhnance the prediction accuracy of coexisting diseases with Hashimoto’s Disease, we propose an algorithm that can create a dynamic system capable of autonomously classifying the diseases that occur together with Hashimoto’s thyroiditis. Our suggested algorithm is novel in that it incorporates correction steps within the training data points. In order to correct misclassified data, we have developed an innovative hybrid algorithm that uses each decision maker’s output as input.

Our algorithm consists of 5 phases, these phases work sequentially to achieve the desired accuracy improvements.

- *Phase 1 (Prepocessing)* This phase includes; data generation, feature extraction and feature selection. All the data were gathered from the datasets that were mentioned in “Dataset preperation procedure” section, then the data was encoded with the k-mer tecnique. Afterwards, feature selecion utilizing LDA tecnique was performed.
- *Phase 2 (Per-sample probablity calculation)* After optaining the transformed data samples, Logistic regressoion was applied to compute the per-sample probabilites.
- *Phase 3 (Clustering of probabilty)* K-means clustering was utilized, the per-sample probabilites defined extra labels based on the clustering. The extra label computation process improves accuracy as it compensates for the effect of the heterogeneous nature of the available data, which consists of documented Hashimoto’s Thyroiditis subsets that vary based on coexisting diseases.
- *Phase 4 (updating labels)* SVM was applied to the updated training data to predict the class labels on test data. labels on the test folder
- *Phase 5 (Final output generation)* Augmented labels were used as input to the designated classifier combination.

In order to offer a more comprehensive illustuation of the method, we firstly mention that the data is initially divided into training and test sets.Then we applied dimensionality reduction and also feature normalization on dataset. The hybrid algorithm involves applying classification to the training set and measuring the class probabilities. We used the K-means clustering algorithm to determine how many classes obtainined the suitable number of class dynamicly and obtained this process resulted in finding the new training labels.

Subsequently, the origanal labels and the labeles that where forecasted by the logistic regression and K-means process were combined. SVM was then applied to the newly generated training data to forecast the class labels on the test set.

Finally, we applied single and ensemble prediction models on classification techniques such as SVM, random forest (RF), logistic regression (LR), K-nearest neighborhood (KNN), and multilayer perceptron (MLP) to each cluster separately to predict concurrent diseases with regard to Hashimoto’s Disease. Our proposed hybrid algorithm enables the categorization of concurrent diseases. We compared the performance of our novel hybrid classification model with that of single and combination classifiers (SVM, RF, LR, KNN, and MLP) in terms of precision, f-measure, sensitivity, and accuracy. K-fold cross-validation was used separately for each cluster to determine the best combination of ensemble classification method parameters. We set the k value to 5. In conjuction hold-out cross validation method was also applied with 60% of the data going to the training set and 40% going to the test set. k-fold cross-validation was utilized to determine the ideal classification technique parameters. Similarly, the dataset was splited into a training set and a test set using hold-out cross-validation. This split was utilized to create a balanced relationship between the size of the training set for model training and the size of the test set for evaluation. To further ensure that the distribution of instances throughout the training and test sets was random, randomization was applied partitioning phase. Data subsets can be randomly rearranged in order to minimize biases and maintain representativeness. It is significant to highlight that we did not employ stratified sampling in this study because the dataset did not reveal a significant number of classes or a big class imbalance, both of which would have necessitated rigorous stratification considerations.

**Fig. 1:**
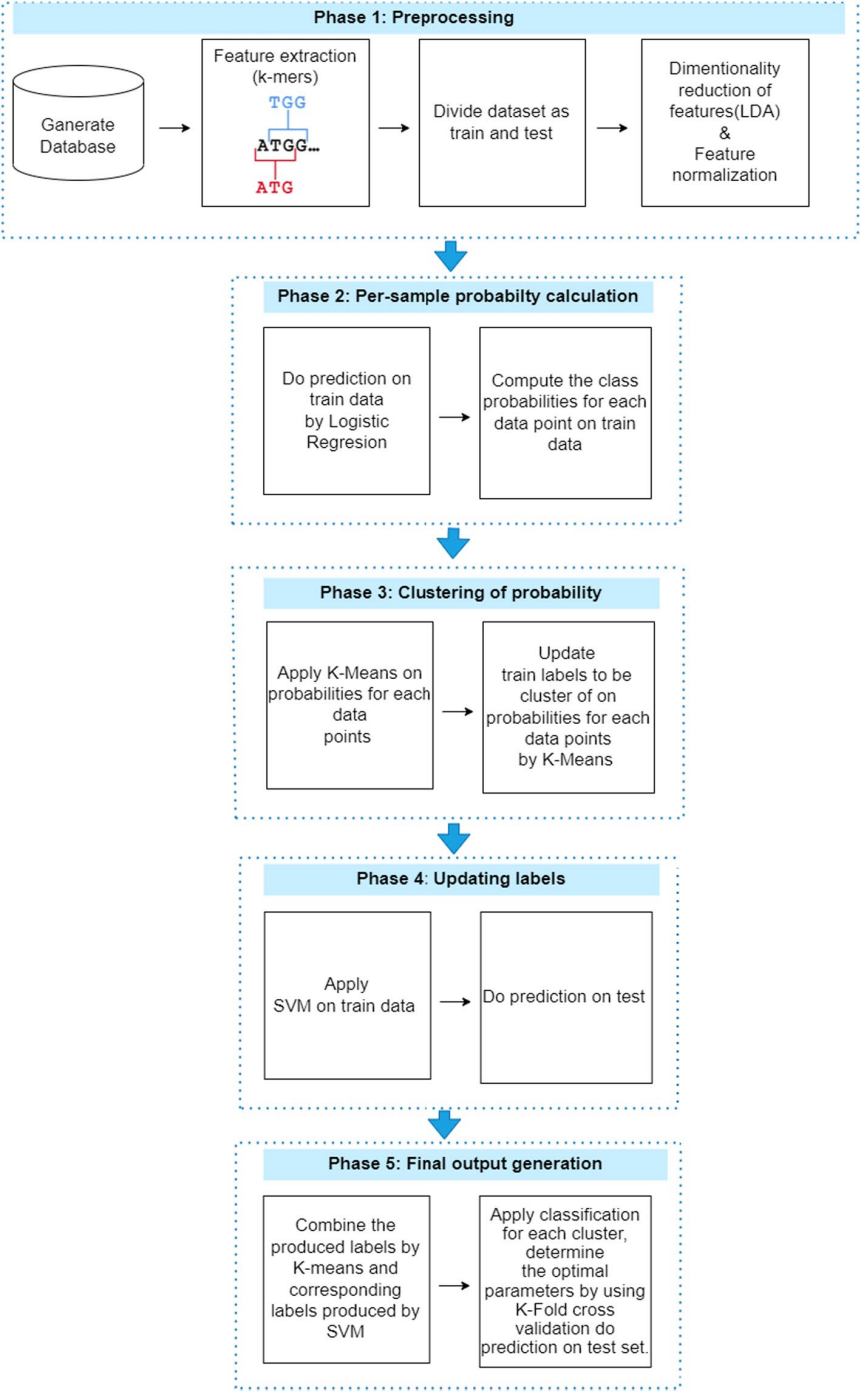
Flow chart of the our hybrid classification algorithm

Overall, K-means and logistic regression were integrated to identify the class labels for each sample in the data set. Then, using a distinct run for each method, SVM classification was applied to each cluster in order to make a final prediction. The flowchart of our proposed model is shown in Fig. 1.

We utilized Python 3 to build our hybrid model by running a Jupyter notebook that was installed by Anaconda Navigator. In this study, we used a variety of Python modules and techniques for feature extraction. We specifically used the “argparse,” “re,” “sys,” “os,” and “platform” libraries, as well as a number of their functions. For a variety of tasks, including classification, clustering, and other related procedures, the scikit-learn package was applied, a popular machine learning library in Python. We were given a complete collection of tools, methods, and functions for machine learning tasks by Scikit-learn. The software provided a dependable and effective interface for applying and assessing various models and methodologies.

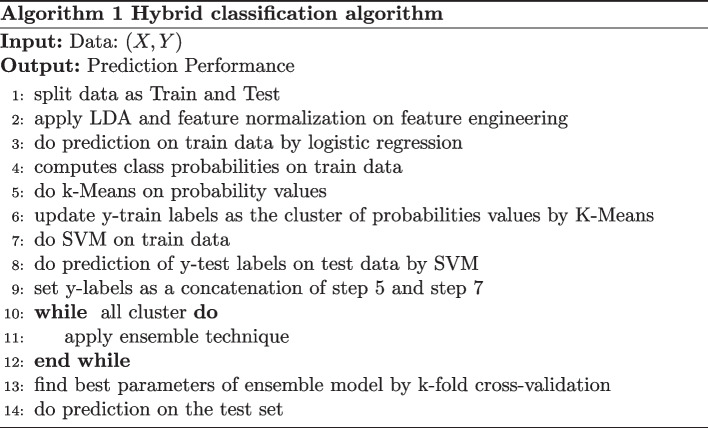



#### Hyperparameter tuning

Bayesian optimization was chosen because of several aspects that made it a good fit for our particular model and dataset. Bayesian Optimization is effective in high-dimensional search spaces. Second, non-linear and non-convex search spaces, which are frequent in machine learning models, can be handled via Bayesian optimization. Bayesian optimization can efficiently direct the search towards optimal hyperparameter configurations even in complex search spaces by using a probabilistic model to describe the underlying function. Third, exploration and exploitation are balanced through Bayesian optimization. It systematically investigates various hyperparameter setups while making use of the potential regions that have performed well. The last reason, Bayesian optimization optimizes the hyperparameters by iteratively evaluating a limited number of configurations. It utilizes a probabilistic surrogate model to approximate the performance of different configurations, reducing the number of actual model evaluations. This leads to efficient utilization of computational resources while achieving good performance.

We used the popular scikit-optimize (skopt) module to create Bayesian optimization in Python. A simple user interface for doing Bayesian optimization is provided by this software.

#### Model evaluation

The performance of our model and other current models is assessed in this study using the following five widely-used measures: precision (p), f-measure (F1) sensitivity and accuracy.

1 $$\begin{aligned} Accuracy= & {} \frac{TP+TN}{TP+TN+FP+FN} \end{aligned}$$

2 $$\begin{aligned} Precision= & {} \frac{TP}{TP+FP} \end{aligned}$$

3 $$\begin{aligned} Sensitivity= & {} Recall = \frac{TP}{TP+FN} \end{aligned}$$

4 $$\begin{aligned} F1= & {} \frac{2*Precision*Recall}{Precision+Recall} = \frac{2*TP}{2*TP+FP+FN} \end{aligned}$$

where the numbers of true positive, false negative, true negative, and false positive samples are denoted by TP (true positive), FN (false negative), TN (true negative), and FP (false positive). In binary classification, the accuarcy describes how the model performs across two classes. sensitivity respectively, show how accurately the predictor was able to anticipate the results of positive and negative samples.The F1-score is a performance metric that weighs recall and precision equally. It is the harmonic mean of recall and precision, offering a single metric to assess how well they are balanced. F1-score has a range of 0 to 1, with 1 denoting exceptional precision and recall and 0 denoting subpar performance in both categories. In binary classification jobs where striking a compromise between recall and precision is crucial, it is frequently utilized. A model’s precision is a metric that assesses how accurately it predicts outcomes that are favorable. Out of all the positive predictions made by the model, it calculates the percentage of real positive forecasts. Precision is concerned with how well the model can reduce false positives, or instances where a result is projected to be positive but is actually negative. A high precision suggests a low rate of false positives for the model, indicating a higher degree of trust in the positive predictions. In general, the higher the aforementioned criteria are, the better the model is at making predictions.

The necessity to completely assess the hybrid classification method’s performance in correctly identifying DNA data is what led to the choice of these performance indicators. Recall or sensitivity quantifies the capacity to correctly detect positive cases, while precision measures the capacity to reduce false positives. F-measure integrates both metrics into a single, well-rounded assessment. In DNA data analysis, where misclassification can have substantial repercussions, these measures jointly describe the model’s capacity to minimize false positives and false negatives. Additionally, accuracy complements the other metrics by taking into account the percentage of correctly identified occurrences to provide an overall assessment of how accurate forecasts were. We aim to present a thorough and nuanced evaluation of the hybrid classification method’s performance using precision, recall, F-measure, sensitivity, and accuracy, making sure that both the capacity to recognize positive instances and the general correctness of predictions are adequately assessed within the particular context of DNA data classification. Precision, recall, F-measure, sensitivity, and accuracy have all been used frequently in the literature in various studies as performance metrics for categorization tasks [65–68]. These measures are frequently employed in academic studies to assess the potency and efficiency of categorization models.

## Experimental results

The provided dataset consists of gene data for patients with autoimmune thyroid disease (AITD) and additional autoimmune disease. This data was obtained from the PUBMED and OMIM databases, and the genes were retrieved from the NCBI database in the fasta format. In order to classify the genes associated with Hashimoto’s thyroid (HT) and other concordant diseases, we used five different algorithms; those are SVM, RF, LR, KNN, MLP. This hybrid algorithm was run with each double, triple, and quadruple combination to determine the best accuracy. The results of our analysis, which are presented in Tables 2, 3, 4, and 5, indicate that combinations of these algorithms had accuracies of over 70$\%$, and the highest attained accuracies are indicated in bold typeface. The results of this analysis showed that our hybrid method achieved the best accuracy for all combinations.

**Fig. 2:**
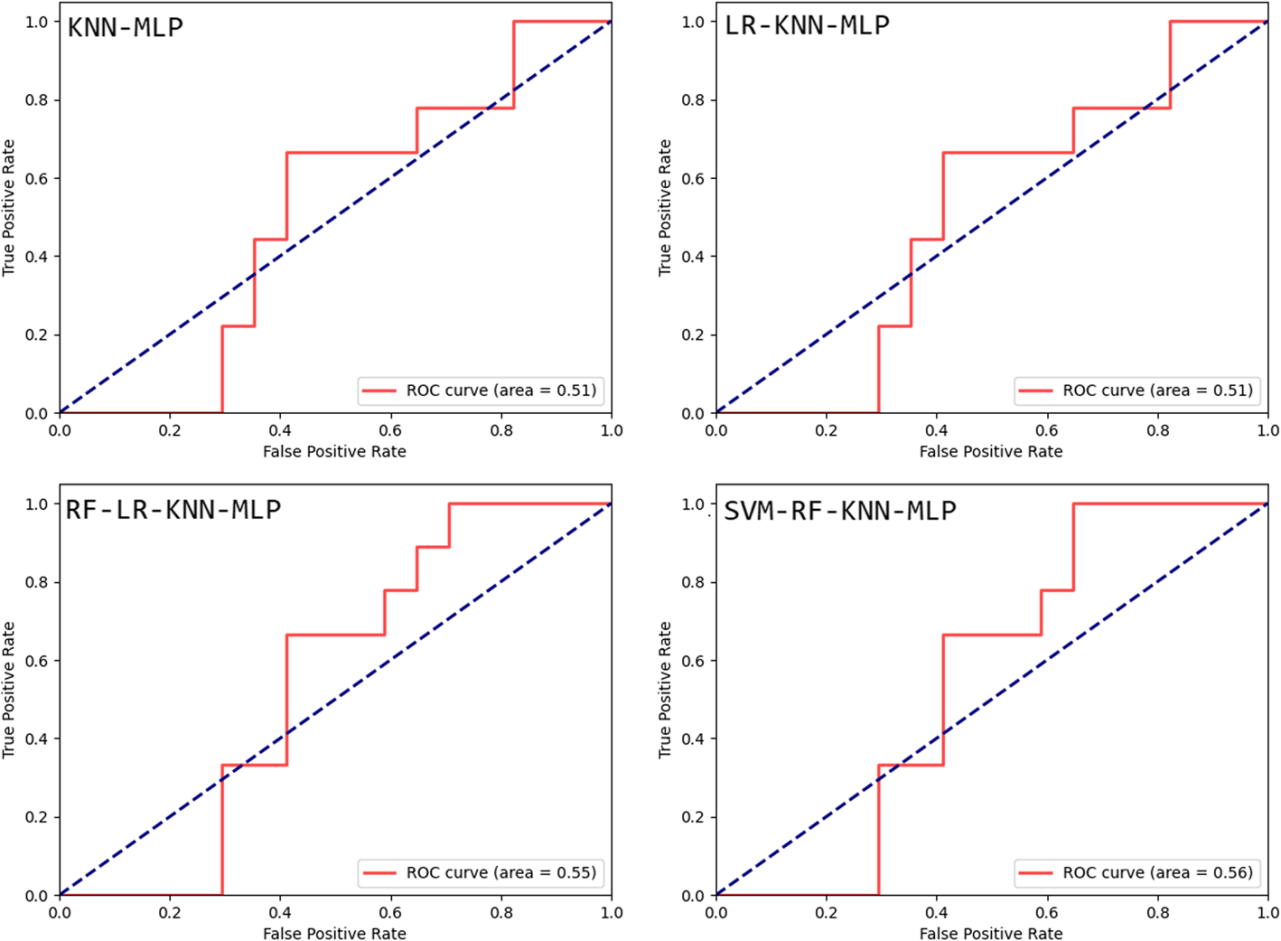
ROC Curves for tested hybrid classification algorithm

We also applied our hybrid algorithm to predict autoimmune diseases that may be associated with HT. The best-performing hybrid method, with RF-LR-KNN-MLP, had an accuracy of 81$\%$. and a sensitivity of 1.0. We ensured the precision of our resulting model to improve the accuracy of our predictions. The ROC curves of our best results from the combinations with our hybrid method are as shown in Fig. 2.

**Table 2:** Results of binary combinations of classification models

Model	Precision (p)	Recall (r)	F-measure (F)	Sensitivity	Accuracy
SVM-RF	0.514	0.9	0.645	0.9	0.486
SVM-LR	0.547	1.0	0.705	1.0	0.547
SVM-KNN	0.665	1.0	0.785	1.0	0.714
SVM-MLP	0.547	1.0	0.705	1.0	0.547
RF-LR	0.514	0.9	0.645	0.9	0.482
RF-KNN	0.662	1.0	0.792	1.0	0.710
RF-MLP	0.514	0.9	0.645	0.9	0.480
LR-KNN	0.667	1.0	0.791	1.0	0.711
LR-MLP	0.54	1.0	0.705	1.0	0.547
KNN-MLP	0.666	1.0	0.796	1.0	**0.735**
Hybrid-KNN-MLP	0.701	1.0	0.802	1.0	**0.786**

**Table 3:** Results of triple combinations of classfication models

Model	Precision (p)	Recall (r)	F-measure (F)	Sensitivity	Accuracy
SVM-RF-LR	0.514	0.9	0.645	0.9	0.485
SVM-RF-KNN	0.654	0.95	0.771	0.95	0.680
SVM-RF-MLP	0.54	0.9	0.659	0.9	0.547
SVM-LR-KNN	0.667	1.0	0.791	1.0	0.714
SVM-LR-MLP	0.547	1.0	0.705	1.0	0.547
SVM-KNN-MLP	0.661	1.0	0.790	1.0	0.712
RF-LR-KNN	0.671	1.0	0.778	1.0	0.721
RF-LR-MLP	0.514	0.9	0.645	0.9	0.480
RF-KNN-MLP	0.668	1.0	0.789	1.0	0.715
LR-KNN-MLP	0.671	1.0	0.793	1.0	**0.726**
Hybrid-LR-KNN-MLP	0.722	1.0	0.823	1.0	**0.792**

**Table 4:** Results of quadruple combinations of classfication models

Model	Precision (p)	Recall (r)	F-measure (F)	Sensitivity	Accuracy
SVM-RF-LR-KNN	0.654	0.95	0.771	0.95	0.677
SVM-RF-LR-MLP	0.547	0.9	0.659	0.9	0.514
SVM-RF-KNN-MLP	0.654	0.95	0.771	0.95	0.681
SVM-LR-KNN-MLP	0.667	1.0	0.798	1.0	0.720
RF-LR-KNN-MLP	0.694	1.0	0.796	1.0	**0.778**
Hybrid-RF-LR-KNN-MLP	0.731	1.0	0.800	1.0	**0.815**

**Table 5:** Results of quinary combinations of classfication models

Model	Precision	Recall	F-measure	Sensitivity	Accuracy
SVM-RF-LR-KNN-MLP	0.623	0.97	0.765	0.95	0.702
Hybrid-SVM-RF-LR-KNN-MLP	0.647	0.97	0.778	0.95	**0.720**

*Z*-test is typically used to determine whether two categorization algorithms produced results with equivalent accuracy.A non-parametric statistical test known as the $x^2$-test can be used to examine if two or more sample classifications are independent of one another. To determine if the two categorization results were statistically different or not, the *Z*-test and $x^2$-test were applied. The *Z*-test was used to interpret the test findings; for instance, a value *Z* $> |$1.96| denotes a statistically significant difference in classification accuracy at the 95% confidence level. According to the statistical analysis of the categorization findings, all of the combinations produced *Z* values more than 1.96. This indicates that significant differences between all of the combinations were discovered. At the 95% confidence level, it was observed that the *Z* value was smaller than 1.96. Notably, the suggested hybrid algorithm outperformed every competing method in terms of classification accuracy. Tables 6, 7, 8, and 9 provides the *Z*-test and $x^2$-test scores for pairwise comparisons between the categorization algorithms. When we compare the t-test results for all tables, they are all greater than the critical value of 3.841, this shows us that combinations of classification accuracy results were found to be statistically different from the proposed hybrid classification algorithm.

**Table 6:** Statistical significance of differences in classification accuracy between binary combination of classfication algorithms and proposed Hybrid algorithm

Classification methods	Proposed hybrid method	*Z*-test	$x^2$-test	*p* value
SVM-RF	Hybrid KNN-MLP	7.02	49.28	$<0$.0001
SVM-LR	Hybrid KNN-MLP	5.72	32.71	$<0$.0001
SVM-KNN	Hybrid KNN-MLP	12.75	162.56	$<0$.0001
SVM-MLP	Hybrid KNN-MLP	8.98	80.64	$<0$.0001
RF-LR	Hybrid KNN-MLP	6.72	45.16	$<0$.0001
RF-KNN	Hybrid KNN-MLP	3.24	10.50	$=0$.0007
RF-MLP	Hybrid KNN-MLP	8.1	65.61	$<0$.0001
LR-KNN	Hybrid KNN-MLP	7.65	58.52	$<0$.0001
LR-MLP	Hybrid KNN-MLP	8.72	76.03	$<0$.0001
KNN-MLP	Hybrid KNN-MLP	4.01	16.1	$<0$.0001

**Table 7:** Statistical significance of differences in classification accuracy between triple combinations of classfication algorithms and proposed Hybrid algorithm

Classification methods	Proposed hybrid method	*Z*-test	$x^2$-test	*p* value
SVM-RF-LR	Hybrid LR-KNN-MLP	5.73	32.8	$<0$.0001
SVM-RF-KNN	Hybrid LR-KNN-MLP	8.88	80.0	$<0$.0001
SVM-RF-MLP	Hybrid LR-KNN-MLP	16.75	280.56	$<0$.0001
SVM-LR-KNN	Hybrid LR-KNN-MLP	5.99	35.88	$<0$.0001
SVM-LR-MLP	Hybrid LR-KNN-MLP	7.2	51.84	$<0$.0001
SVM-KNN-MLP	Hybrid LR-KNN-MLP	12.4	153.76	$<0$.0001
RF-LR-KNN	Hybrid LR-KNN-MLP	3.2	10.34	$=0$.0009
RF-LR-MLP	Hybrid LR-KNN-MLP	15.04	225.5	$<0$.0001
RF-KNN-MLP	Hybrid LR-KNN-MLP	8.72	76.03	$<0$.0001
LR-KNN-MLP	Hybrid LR-KNN-MLP	11.02	121.44	$<0$.0001

**Table 8:** Statistical significance of differences in classification accuracy between quadruple combinations of classfication algorithms and proposed Hybrid algorithm

Classification methods	Proposed hybrid method	*Z*-test	$x^2$-test	*p* value
SVM-RF-LR-KNN	Hybrid RF-LR-KNN-MLP	14.6	213.1	$<0$.0001
SVM-RF-LR-MLP	Hybrid RF-LR-KNN-MLP	7.81	61.0	$<0$.0001
SVM-RF-KNN-MLP	Hybrid RF-LR-KNN-MLP	17.25	306.25	$<0$.0001
SVM-LR-KNN-MLP	Hybrid RF-LR-KNN-MLP	8.9	79.21	$<0$.0001
RF-LR-KNN-MLP	Hybrid RF-LR-KNN-MLP	11.8	139.24	$<0$.0001

**Table 9:** Statistical significance of differences in classification accuracy between quinary combinations of classfication algorithms and proposed Hybrid algorithm

Classification methods	Proposed hybrid method	*Z*-test	$x^2$-test	*p* value
SVM-RF-LR-KNN-MLP	Hybrid-SVM-RF-LR-KNN-MLP	6.54	42.77	$<0$.0001

**Fig. 3:**
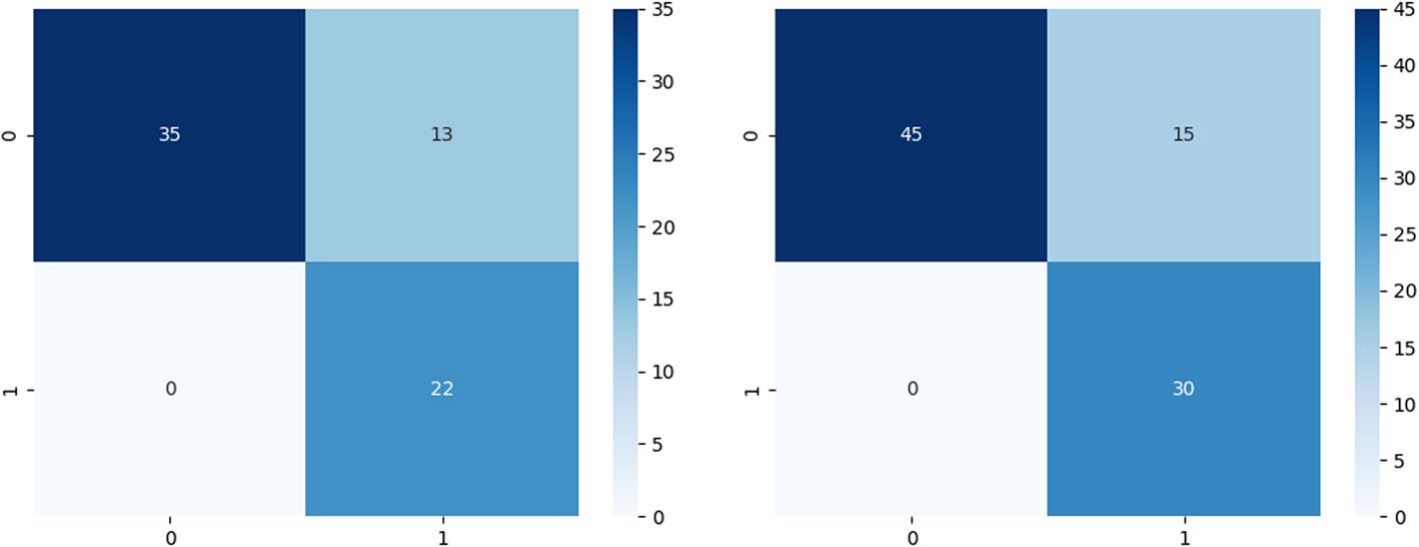
Confusion matrix for the best hybrid classification combination (Hybrid-RF-LR-KNN-MLP) on the test set (left), Confusion matrix for the best hybrid classification combination (Hybrid-RF-LR-KNN-MLP) on the train set (right)

The confusion matrix of the test data for this particular instance shows that the model accurately identified 35 cases as genuine positives and 22 instances as true negatives. Additionally, it wrongly predicted 13 cases as false positives while properly predicting none as false negatives. These numbers show how well the model can distinguish between positive and negative occurrences in the dataset. The confusion matrix for these training examples shows that the model correctly identified 45 instances as genuine positives and 30 instances as true negatives. Additionally, it wrongly labeled 15 occurrences as false positives while accurately predicting no instances as false negatives. These results show how the model can successfully locate both positive and negative examples in the dataset Fig. 3. It is significant to emphasize that the implications of the confusion matrix results, together with domain-specific factors and research goals should be taken into account within the specific context of the classification task.

**Fig. 4:**
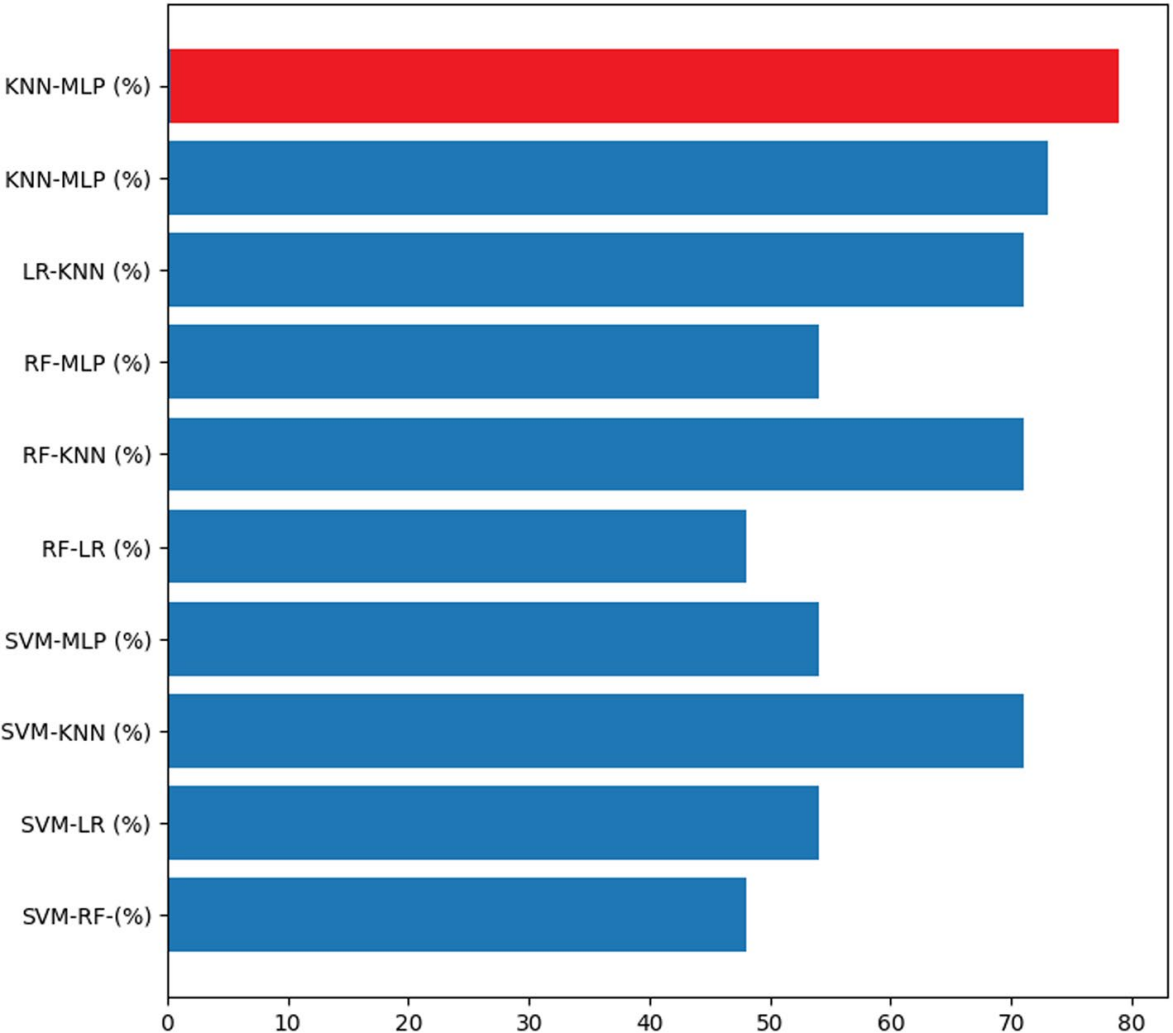
Accuracy result for binary combination

**Fig. 5:**
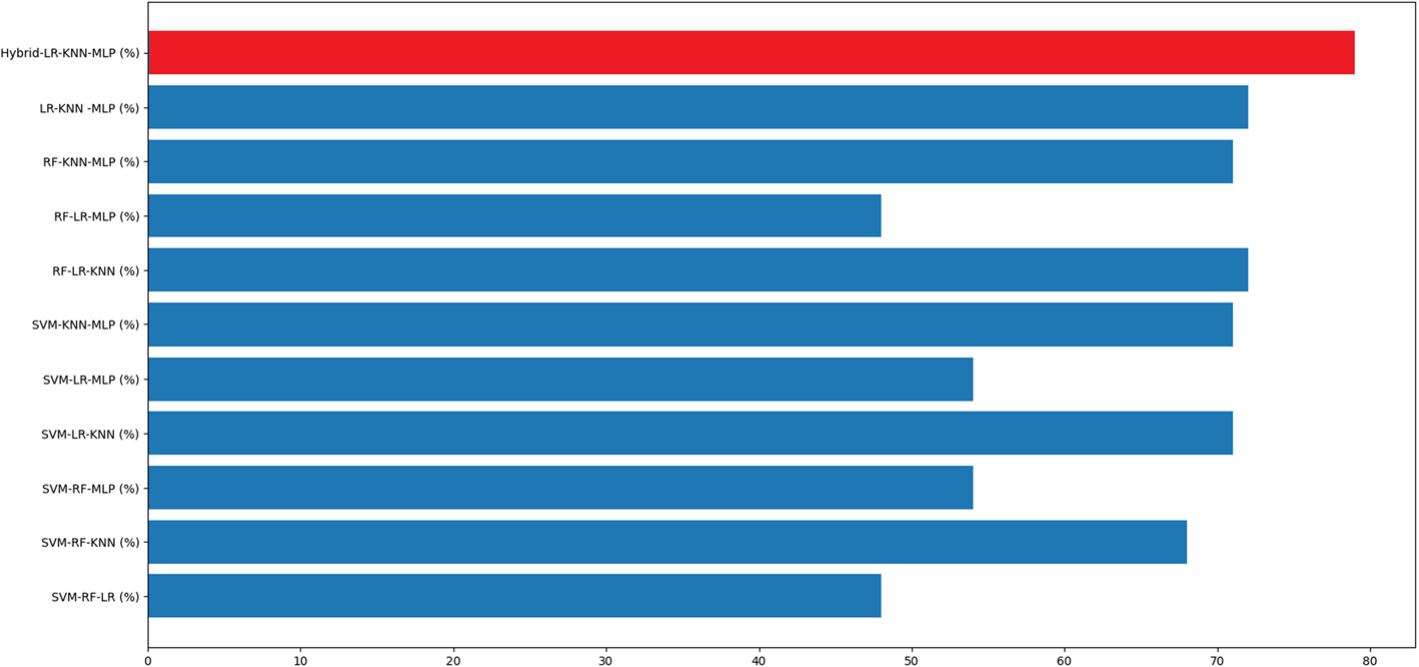
Accuracy result for triple combination

**Fig. 6:**
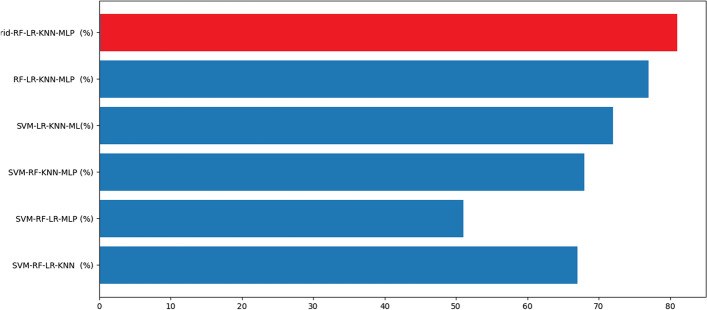
Accuracy result for quadruple combination

**Fig. 7:**
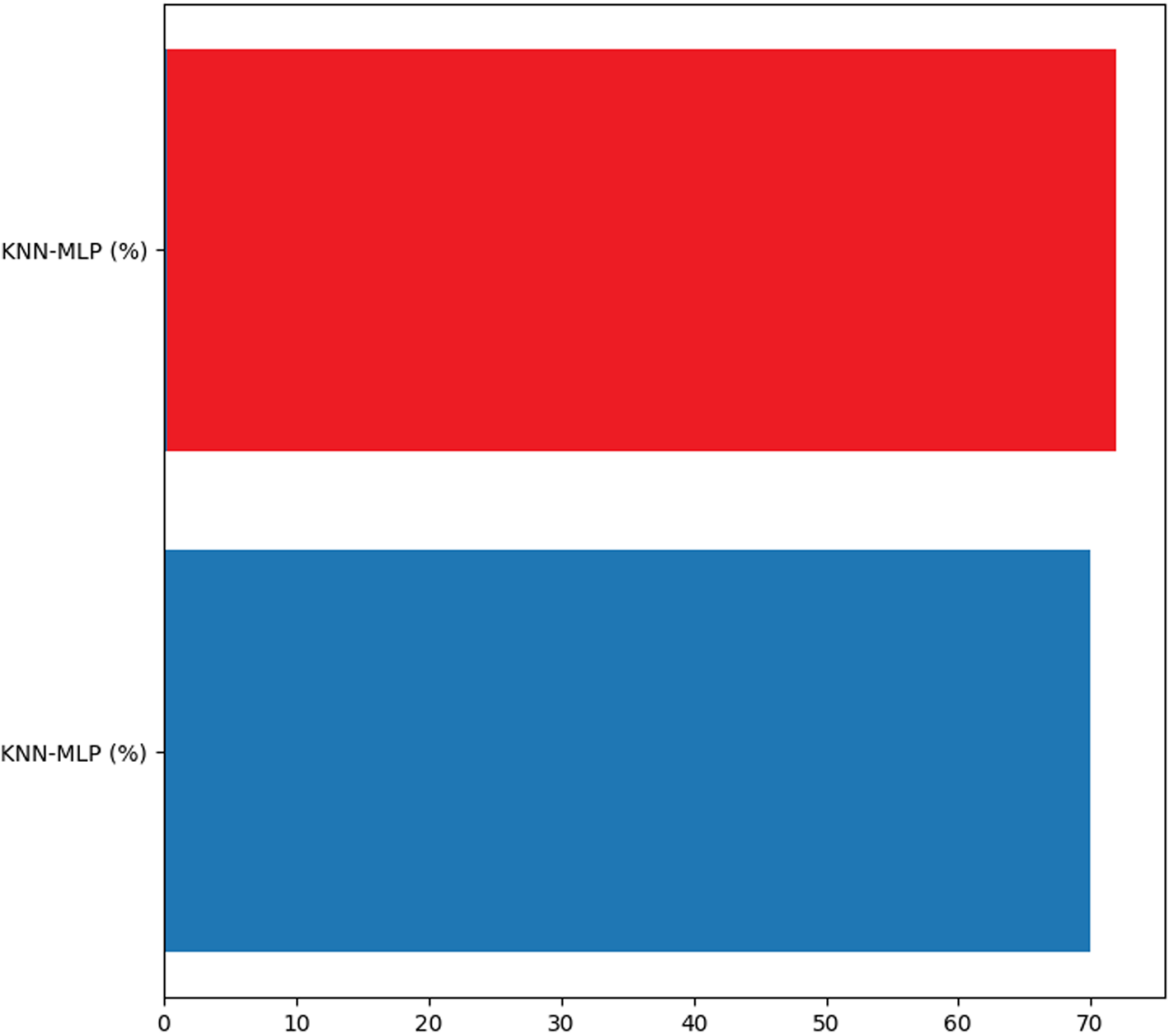
Accuracy result for quinary combination

The graphs in Figs. 4, 5, 6 and 7 present the accuracy percentages obtained for each combination. Upon visual examination of the graphs, it is evident that the hybrid method consistently achieves a significantly higher level of accuracy.

The precision-recall curve graph offers a helpful visual picture of the model’s performance, with accuracy on the y-axis and recall on the x-axis. Fundamental metrics for classification tasks include recall and precision. Recall measures the capacity to properly identify positive occurrences, while precision measures the accuracy of positive predictions. This graph compares recall and precision to highlight how these two metrics trade-off as the categorization threshold is changed. We may evaluate the model’s performance at various operating points and choose the best compromise between precision and recall depending on their particular needs by evaluating the curve. The graph of the precision-recall curve provides a thorough analysis of the model’s performance at various recall levels. This enables researchers to weigh the performance trade-offs of the model and understand how changes in the classification threshold affect precision and recall.

**Fig. 8:**
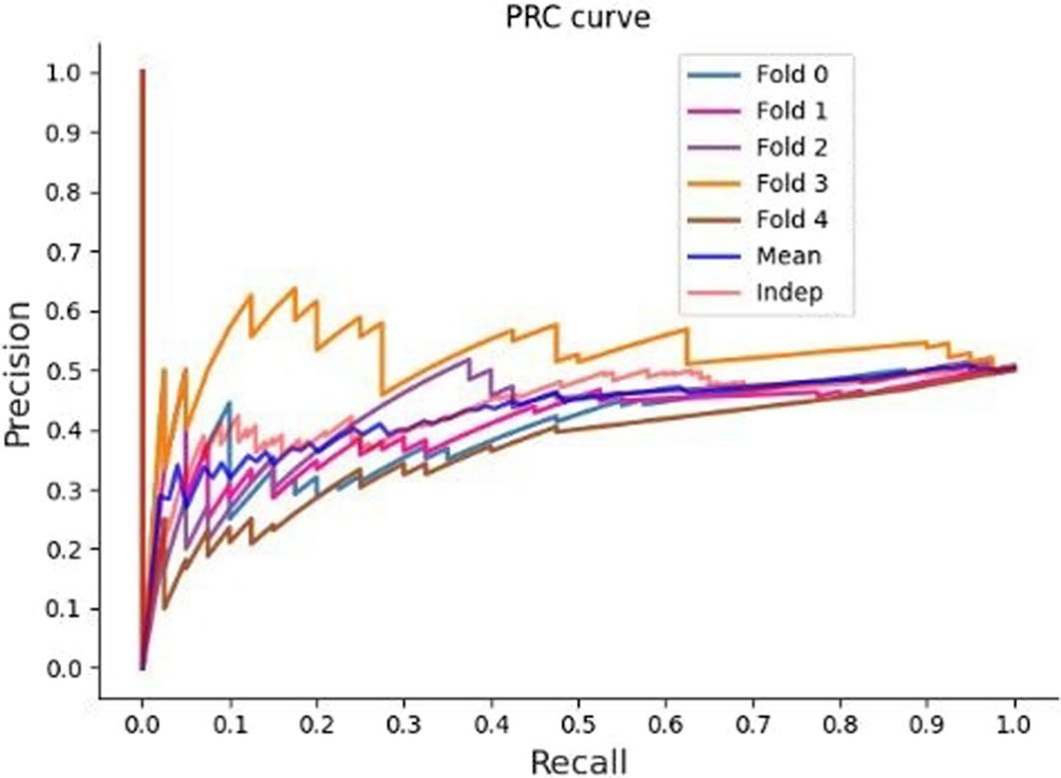
Precsion Curve graph for the best hybrid classification combination (Hybrid-RF-LR-KNN-MLP)

It is important to take into account the contextual aspects of the classification task, such as the dataset’s features, class distribution, and research goals, while interpreting the precision-recall curve graph. For each fold in cross-validation, there exists a specific precision-recall graph as shown in Fig. 8.

## Discussion

Hashimoto’s thyroiditis (HT) is an autoimmune thyroid disease characterized by diffuse lymphocytic infiltration in the thyroid gland [69]. Its prevalence in the population is 2$\%$, with a gradual increase over time, and an incidence rate of 0.3$-$1.5$\%$ per year. HT is commonly observed in women between the ages of 30–50 and has familial characteristics. The disease is detected in 95$\%$ of women, 8–10 times more frequently than in men [70]. HT is often associated with other autoimmune diseases, such as Graves disease, Type 1 Diabetes Mellitus, Autoimmune orchitis or oophoritis, idiopathic hypoparathyroidism, lymphocytic hypophysitis, non-endocrine, organ-specific autoimmune diseases, Pernicious anemia, vitiligo, rheumatoid arthritis, idiopathic thrombocytopenic purpura, myasthenia gravis, Sjogren’s syndrome, chronic active hepatitis, systemic lupus erythematosus, Primary biliary cirrhosis, Renal tubular acidosis, Down syndrome, Turner syndrome, and Klinefelter syndrome [71].

In the health sector, it is essential to utilize health management for effective health services, including disease diagnosis, treatment, rehabilitation, disease prevention, and improvement of the overall health level of the society, especially considering the increasing elderly population and the incidence of chronic diseases [72]. New technologies such as machine learning techniques and artificial intelligence applications can be employed to aid in disease classification. Our hybrid classification method was able to find the appropriate algorithm for the accurate classification of HT-related diseases. Using fasta data, experiments enabled an expanded understanding of genes relate to concomitant autoimmune diseases.

Our proposed hybrid classification technique achieved, an accuracy of over 70$\%$. Our study provides insights into the relationship between thyroid autoimmune diseases and other comorbid diseases using gene sequence data with various features and various variety of hybrid classification methods. Our algorithm successfully dealt with the problems involved with categorizing concomitant disorders associated with Hashimoto’s thyroiditis by using a dynamic decision-making process and using the particular features of our benchmarking dataset.

Our hybrid model performed better than conventional machine learning methods alone and in combination, illustrating its potency in dealing with the genetic variability seen in complex disorders. This exceptional success can be attributed to the model’s capacity to represent complex interdependencies and interactions between genes and diseases, enabling more precise and accurate categorization outputs. Moreover, the enhanced functionality of our hybrid model has useful implications for the management and diagnosis of autoimmune disorders. The result of our proposal opens the door for futher development of focused treatment interventions as well as clinical decision-making could all benefit from its robustness and precision. We highlight the major advancements made possible by our new approach by offering a thorough review and comparison of our model to previous approaches. Our hybrid model performs better than other methods when it comes to correctly diagnosing concurrent disorders that are linked to Hashimoto’s thyroiditis. The steady and noticeable gains made in a range of performance indicators highlight the potential value of our model for clinical use, genetic research, and the creation of new medicines. The potential uses of our study in the creation of genetic screening instruments and lab tests that specifically target genetic risk factors. The classification capabilities the hybrid algorithm can be used by genetic screening tools to identify people who are at risk for co-occurring diseases with Hashimoto’s thyroiditis as a result of our study. Based on DNA sequences, many disorders may be accurately categorized, which can help with early detection and intervention and improve patient outcomes. Furthermore, the development of focused laboratory research can benefit from the discovery of genetic risk factors linked to Hashimoto’s thyroiditis and the accompanying disorders. These studies could concentrate on figuring out the disease’s underlying biological causes and identifying viable treatments.

Using the hybrid algorithm, for instance, to check people with Hashimoto’s thyroiditis for the existence of particular genetic variants linked to a higher vulnerability to particular concomitant conditions, is one example of a use-case scenario. The identified people can subsequently be a part of laboratory studies designed to comprehend the underlying biological mechanisms and create individualized treatment plans.

The integration of our hybrid approach with already available clinical diagnostic tools could be another use-case scenario. Healthcare practitioners can improve their diagnostic precision and offer more individualized treatment strategies for patients with Hashimoto’s thyroiditis and its accompanying disorders by using DNA sequence analysis and categorization results. Our study has the potential to be used in creating genetic screening tools and running lab tests that focus on genetic risk factors. We may provide a clearer and more thorough grasp of the practical consequences and advantages of our research by going into more depth about these applications, including specific use-case scenarios.

The precise results obtained using our novel hybrid method for codominant diseases associated with Hashimoto’s thyroid disease can be similarly applied to other diseases. Further studies using the latest DNA sequence and methods are needed to confirm whether similar results can be obtained from experiments on gene sequence data.

## Conclusions

The study presented in this work offers a unique and innovative approach in two distinct ways. Firstly, we have developed and presented a novel dataset utilizing various sources, which has not previously been established in this particular field. As a result, a fresh benchmarking dataset has been produced. Secondly, leveraging this dynamic dataset as a guide, we proposed an alternative strategy for a more practical and efficient solution, referred to as the “hybrid classification model”. This model comprises two stages: in the first stage, the training labels on the probability of class vector, which are frequently utilized for genetic data prediction, are updated through K-means clustering. In the second stage, the updated training dataset and test dataset are classified and combined using the support vector machine (SVM) algorithm. The final stage involves predicting the concomitant diseases in each cluster using various combinations of Random Forest (RF), Logistic Regression (LR), K-Nearest Neighbors (KNN), and Multi-Layer Perceptron (MLP) allowing for comparison. The precision recall, f-measure, sensitivity, and accuracy of each of these combinations are contrasted with the hybrid variants, we have demonstrated that the hybrid base classification method is a viable approach for studying the genetic heterogeneity of complex autoimmune diseases such as Hashimoto’s Thyroiditis (HT) and identifying its concomitance with other autoimmune diseases. These findings are expected to aid physicians in the clinic in predicting the concomitance of HT with other autoimmune diseases.

## Data Availability

The manuscript contains third party material and obtained permissions are available on request by the author.

## Code availability

The source codes of our model is available on request by the author.
